# Pain Interventions for people with dementia: a quasi-experimental study

**DOI:** 10.1186/s12904-022-01118-9

**Published:** 2022-12-29

**Authors:** Frank Spichiger, Thomas Volken, Georg Bosshard, Nicole Zigan, Geneviève Blanc, Andreas Büscher, Martin Nagl-Cupal, Mathieu Bernard, Eve Rubli Truchard, Philip Larkin, Andrea Koppitz

**Affiliations:** 1 HES-SO, School of Health Sciences Fribourg, Institute of Applied Health Research, Route des Arsenaux 16a, 1700 Fribourg, Switzerland; 2 Lausanne University Hospital and University of Lausanne, Institute of Higher Education and Research in Healthcare, Route de la Corniche 10, Lausanne, 1010 Switzerland; 3 ZHAW School of Health Sciences, Katharina-Sulzer-Platz 9, 8400 Winterthur, Switzerland; 4 Alters- und Pflegezentrum Bruggwiesen, Märtplatz 19, 8307 Effretikon, Switzerland; 5 Berner Bildungszentrum Pflege, Freiburgstrasse 133, 3008 Bern, Switzerland; 6 Osnabrück University of Applied Sciences, Faculty of Business Management and Social Sciences, Caprivistraße 30A, 49076 Osnabrück, Germany; 7 University of Vienna, Institute of Nursing Science, Alser Strasse 23, 1080 Vienna, Austria; 8 Lausanne University Hospital and University of Lausanne, Palliative and Supportive Care Service, Avenue Pierre-Decker 5, 1011 Lausanne, Switzerland; 9 Lausanne University Hospital and University of Lausanne, Service of Geriatric Medicine and Geriatric Rehabilitation, Avenue Pierr-Decker 5, 1011 Lausanne, Switzerland

**Keywords:** People with dementia, Pain management, Nursing home, Work-based learning, Work-related learning

## Abstract

**Background:**

Due to the complexity of the provision of care for people with dementia, pain assessment and management is still considered to be lacking. An optimal way to support frontline staff in providing pain assessment and management for people with dementia living in nursing homes has not yet been identified. The success of supporting interventions seems dependent on contextual factors in the nursing homes. This study, therefore, analyzes the feasibility of a nurse-led training intervention, using repeated on-site case studies, in modifying pain intensity and frequency in people with dementia.

**Methods:**

Using a quasi-experimental design, we undertook a multi-center study of nurse-led training in pain management, with subsequent on-site case studies. Healthcare workers from 3 nursing homes assessed pain in 164 residents with dementia over 147 days. We used mixed-effect growth curve models with spline regression to analyze the data.

**Results:**

We found that on-site case studies support frontline staff with pain management and assessment. Repeated reflection in case studies led to significantly longer pain free intervals (from 4.7 at baseline to 37.1 days at second follow-up) and decreased frequency of pain events (OR 0.54 at first follow-up and 0.43 at second follow-up). However no trends regarding pain intensity could be found. Therefore, on-site case studies may be valuable for improving pain frequency and pain-free intervals over time.

**Conclusion:**

This feasibility study shows the potential of on-site support for frontline nursing home staff. On-site case studies may also affect health outcomes in people with dementia. However, the complexity of dementia care necessitates the management of a broader range of needs.

**Trial registration:**

The study was retrospectively registered on the tenth of January 2017 with the German registry of clinical trials (DRKS00009726).

## Background

In nursing homes, 60–80% of people with dementia experience pain regularly [1]. As such, pain management for people with dementia often becomes a trade-off between treating pain, adressing the security concerns of the nursing home staff, and avoiding pain exacerbation [2]. Additional barriers to the treatment of pain in people with dementia living in nursing homes are identified by two main factors [1]: the suitable administration of pharmacological and non-pharmacological interventions, and difficulties in interprofessional collaboration in planning care between family members, frontline staff, physicians, and other nursing home staff. Nursing and medical care for people with dementia living in Swiss nursing homes is delivered not only by registered nurses but also health care associates, interns and vocationally trained nurses [3] which are referred to as frontline staff. Frontline staff in nursing homes, who are responsible for symptom management, often lack the knowledge and time to engage in symptom identification and management. Gilmor-Bykovsky et al. [4] describe a model for the behaviour of frontline staff regarding their responses to suspected pain in people with dementia - the “response certainty of pain” model. In everyday care, frontline staff experiences varying degrees of certainty regarding suspected pain. Especially when initially suspecting pain in people with dementia, frontline staff tends to conceptualize suspected pain as a change in behaviour. These situations are seldom responded to with pain relief intervention trials [4], despite current guidance and evidence [5].

Staffing levels, funding, and quality management regulations vary widely among Swiss nursing homes [3]. Continuous staff education in nursing homes poses unique challenges. On one hand, Swiss nursing home staff fluctuation oscillates around 11% [3], rendering one-time educational sessions futile in the long term. On the other hand, as Surr et al. [6] described, instructions and workshops for nursing home staff are usually plagued by absenteeism or staff time restrictions. Moreover, educational interventions for pain management in the form of sessions or workshops have failed to prove their long-term efficacy according to Adam et al. [7] in other settings and Tsai et al. in dementia care [8]. Bedside coaching and interventions may mitigate organizational challenges in the nursing home setting [6]. An on-site, tailored approach may help with knowledge translation from theory to practice [6]. The coaching in pain management for frontline staff should be individually tailored to each institution and the people with dementia’s situations [6], since context and situations can be considered complex [6, 8]. However, follow-up evaluations of the long-term sustainability of educational interventions related to pain management are rarely available [7].

Therefore, we developed and tested the feasibility of a two-phase, nurse-led on-site case study intervention to improve pain management in people with dementia living in nursing home [9].

## Methods

### Study design

This multi-center feasibility study with nurse-led interventions used a quasi-experimental research design. Here we partially report on our trial outlined in the protocol published previously [9]. The process evaluation conducted with qualitative methods is reported separately.

### Setting and sample

We conducted this study at three Swiss nursing homes in the cantons of Zürich and Thurgau. A convenience sample of 164 patients were observed for 147 days over a total study duration of 425 days or until they were censored. People with dementia were recruited for this study if they lived in one of the three nursing homes and had a dementia diagnosis in their records or symptoms of dementia as evidenced by the minimum data set. The minimum data set items: comatose status, short-term memory, ability to make decisions, making self understood and eating performance were assessed [10, 11] with the nursing homes to determine people with dementias’ eligibility for the study.

The intervention was aimed at frontline nursing home staff, including registered nurses and healthcare assistants, interns, and those in other nursing roles. To participate, frontline staff had to be 18 years or older and had to have been employed at the respective nursing home for at least a year prior to the study. Furthermore, they had to be employed at least a part-time equivalent of 30% and adequate spoken and written language skills in order to participate in the study. The previously published study protocol presented the sample-size and statistical power considerations for our event history and multilevel analyses [9].

### Primary endpoints

The pain frequency, pain intensity, pain episode duration, and pain-free intervals—as measured or derived by the “Beurteilung von Schmerz bei Demenz” (BESD; the German version of the Pain Assessment in Advanced Dementia Scale [PAINAD]) scores—were assessed by the participating frontline staff at least once daily, and over 24 h at suspected pain events, during the three 49-day periods [12]. Like with most observational scales for pain assessment in dementia also with BESD, there is no true certainty if the observed individual is in pain or not [13]. BESD demonstrated high levels of inter-rater and retest reliability and was developed for use with patients with advanced dementia [14–16]. It covers five categories to observe (breathing, negative vocalization, body language and consolability) with three manifestations each (e.g. none = 0 to severe = 2) [12]. BESD scores are calculated based on observations during care and range from 0 to 10, with a cut-off at 2 points indicating mild pain [17].

### Data collection

Data were collected by trained frontline staff during all shifts when pain was suspected or at least once a day. They filed their observations during a baseline period (T0) and two follow-up periods (T1 and T2) these are illustrated regarding absolute time in Table 1.

**Table 1:** Study time schedule

Time (days)													
**0**	**49**	**50**	**99**	**100**	**160**	**161**	**210**	**211**	**260**	**261**	**380**	**381**	**430**
Baseline (T0)		Intervention cycle		Observation-free		Follow-up (T1)		Intervention cycle		Observation-free		Follow-up (T2)	

As mentioned above, BESD scores were assessed by the participating frontline staff at least once daily and at suspected pain events. In addition, the sociodemographic data of the people with dementia enrolled in this study were obtained via the minimum data set. Since each observation period spanned 49 days data for 147 days was analyzed.

The two-part intervention was delivered for 49 days after baseline and again after the first follow-up. After the first intervention cycle and before T1, there was an observation-free period of 60 days. There was also an observation-free period of 119 days between the second intervention cycle and T2. Therefore, for each nursing home, the total study duration amounted to 430 days.

### Intervention

The nurse-led intervention was divided into two parts: (1) an on-site training workshop for frontline staff, and (2) on-site case studies and individual coaching with the frontline staff. Intervention elements are detailed in Table 2. The two part intervention was designed by the research team around best practice recommendations for the advanced nursing process [18–20] without user or patient involvement.

**Table 2:** Description of intervention elements

Action	Actor	Context	Target	Time
**Training workshop**				
Training in systematic and structured pain assessment, communication and management with BESD, SOAPIER and ISBAR	Clinical nurse specialists trained in geriatric and palliative care	Nursing home meeting rooms.	Frontline staff (nursing associates, registered nurses)	Before baseline and at the beginning of each intervention cycle
**Pain assessment**				
Identification and assessment of pain using BESD.	Frontline staff (nursing associates, registered nurses)	When pain is suspected during care interactions	People with dementia living in respective nursing homes	While caring for people with dementia
Documentation of BESD observation using SOAPIER	Frontline staff (nursing associates, registered nurses)	Nursing home staff office, during write-up	Registered nurses, intervention nurse	After BESD observation, or at least once daily.
Communication of BESD observation using ISBAR	Frontline staff (nursing associates, registered nurses)	When pain is likely.	Registered nurses, intervention nurse, physicians	After observing BESD total score above cut-of or person with dementia self-reports pain.
Individual coaching on BESD, SOAPIER and ISBAR use.	Clinical nurse specialists trained in geriatric and palliative care	Nursing home units	Frontline staff (nursing associates, registered nurses)	Bi-weekly during both intervention cycles
**Pain management**				
Individual support and advice to develop a pain action plan.	Clinical nurse specialists trained in geriatric and palliative care	Nursing home units	Frontline staff (nursing associates, registered nurses)	Bi-weekly during both intervention cycles
Case studies on pain action plan implementation issues and strategies	Clinical nurse specialists trained in geriatric and palliative care	Nursing home units	Frontline staff (nursing associates, registered nurses)	Bi-weekly during both intervention cycles

Based on Pressaus’ [21] Action, Actor, Context, Target, Time (AACTT) Framework.

*Abbreviations*: *BESD* Beurteilung von Schmerz bei Demenz, *ISBAR* Identify, Situation, Background, Assessment, Recommendation, *M* mean, *N* number, *SOAPIER *Subjective data, Objective data, Assessment data, Plan, Intervention, Evaluation, Recommendation

The two-hour training workshop was conducted on-site by external clinical nurse specialists trained in geriatric and palliative care and aimed to train frontline staff in systematic pain assessment. The on-site coaching was designed to guide the frontline staff in systematic pain management, including systematic charting. The case studies were related to bedside coaching and communication training, to support interdisciplinary communication about pain and symptom management in people with dementia. The on-site team took fifteen to sixty minutes to discuss pain and symptom management and their observations during caregiving with the intervention nurse. Before suggesting prescription changes to the team, the intervention nurse conferred with an on-call geriatrician during the coaching intervention.

### Ethical considerations

The Zürich cantonal ethics committee and the ethics committee of Eastern Switzerland (2016-0001) considered this study at registration a quality improvement project without the need for informed consent. Nonetheless, participating people with dementia and their legal representatives were asked to provide written informed consent. The study was registered with the German registry of clinical trials (DRKS00009726).

### Analysis

We fitted mixed-effect growth curve models with Stata 15.1, using spline regression with five knots to estimate the adjusted course of pain frequency, pain intensity, and pain-free interval duration. Because the data were derived from repeated measures, had multiple levels, and had nested factors (e.g., people with dementia nested in departments, nested in nursing homes), a simpler model may not produce unbiased and precise estimates [22]. The data were checked for linearity, additivity, and normality, and no issues were found. Missing data stemmed from attrition (i.e. deaths) which the models where adjusted for. We adjusted the models for nursing homes, age (centered at the mean), sex, number of previous pain events, and deaths during the study period. To assess the course of pain frequency by nursing home, we included interaction terms based on the spline knots, with the nursing home variable as a fixed effect. In addition, patient-specific random effects were included to capture potentially correlated observations for the same person with dementia (i.e., clustering due to repeated measurement). Similar models, in which splines were replaced with period-specific dummy variables, were estimated for both the baseline and follow-up periods.

## Results

The average age of people with dementia included in this study was 85.5 years (standard deviation [SD] = 8.5 years), and 72% of the participating people with dementia were female. The resulting data set contained 20,084 observations (Table 3). During the study, the attrition rate was 29.9%. In total, 839 observations indicated pain events, and in 73.2% of the people with dementia, pain was observed at least once. On average, the people with dementia experienced 5.3 (± 7.6) pain events per day.

**Table 3:** Sample description

	Nursing home			
	**I**	**II**	**III**	**Total**
Number of people with dementia (n)	46	69	49	164
Age in years (mean [SD])	83.1 [8.2]	86.6 [8.9]	86.2 [8.0]	85.5 [8.5]
Female (n [%])	27 [60.1]	53 [76.8]	37 [75.5]	118 [72.0]
Number of observations (n)	5,576	8,715	5,793	20,084
Attrition / deaths (n [%])	18 [39.1]	19 [27.5]	12 [24.5]	49 [29.9]

Frontline staff sociodemographics were captured at T1 and T2 and are described in Table 4. No data on staff turnover was collected during the study.

**Table 4:** Frontline staff sociodemographics

	Nursing home			
	**I**	**II**	**III**	**Total**
Number of staff (n)	67	85	70	222
Age in years (mean [SD])	45 [12.6]	46.9 [11.4]	46.4 [10.5]	46.4 [11.5]
Work experience (mean [SD])	15.6 [9.2]	16.3 [10.5]	17.4 [11.5]	16.4 [10.4]
Female (n [%])	63 [94]	76 [89.4]	60 [85.7]	199 [89.6]
Registered nurses (n [%])	25 [37.3]	39 [45.9]	24 [34.3]	88 [39.6]
Nursing associate professionals (n [%])	16 [23.9]	10 [11.8]	13 [18.6]	39 [17.6]
Health care assistants (n [%])	26 [38.8]	36 [42.4]	33 [47.1]	95 [42.8]

### Frequency of pain

**Fig. 1:**
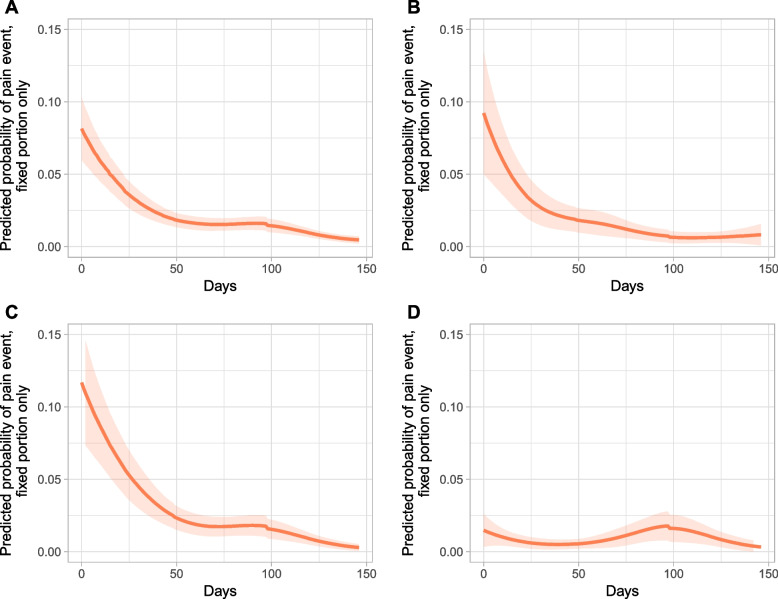
Course of pain event probability by nursing home over 147 days, with 95% confidence interval (CI). **A**: All nursing homes; **B**: nursing home I; **C**: nursing home II; **D**: nursing home III.

The probability of a pain event significantly decreased in all nursing homes over the 147 days of observation. The declining pain event probability was most considerable from the baseline to the first follow-up (T1; Fig. 1). During both follow-ups, the odds of experiencing pain compared to the baseline were significantly lower (*p* < .01), with an odds ratio of 0.54 at T1 and an odds ratio of 0.43 at T2. However, the nursing homes were heterogeneous in the frequency of pain events, even after controlling for the factors described above. The estimated model’s Wald test was significant (*p* < .001), at χ^2^(6) = 132.1.

### Pain intensity

**Fig. 2:**
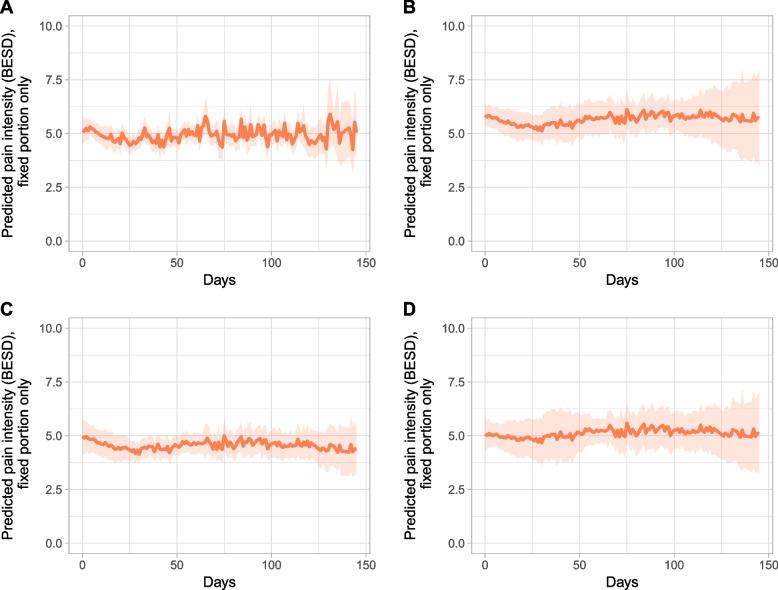
Course of pain intensity (i.e., BESD score) by nursing home over 147 days, with 95% CI. **A**: All nursing homes; **B**: nursing home I; **C**: nursing home II; **D**: nursing home III.

Pain intensity did not show any trends over time, either overall or among individual nursing homes (Fig. 2). The adjusted pain intensity (i.e., BESD score) for people with pain events oscillated around 5 out of a possible 10. Differences between the nursing homes were not significant over time. Furthermore, covariates such as age, sex, frequency of previous pain events, and death during the study showed no significant effect. The estimated model’s Wald test was significant (*p* < .05), at χ^2^(12) = 26.05.

### Pain-free interval duration

**Fig. 3:**
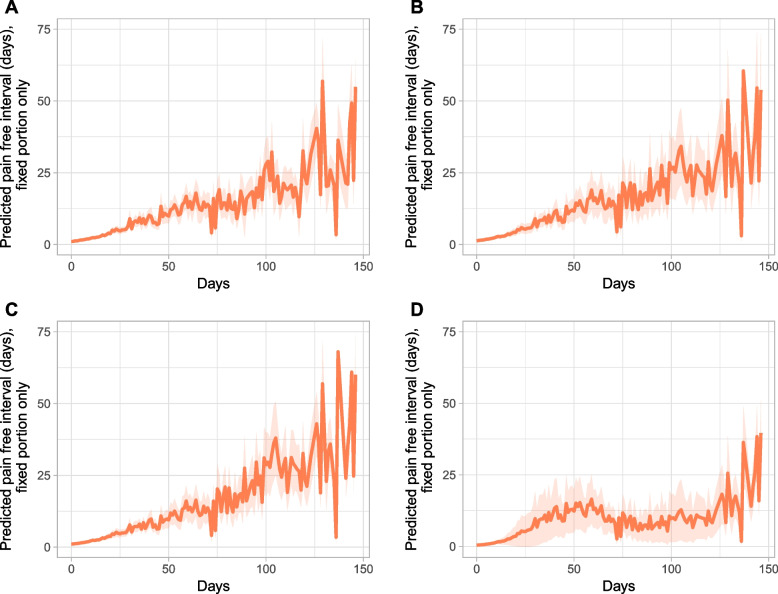
Course of pain-free interval duration by nursing home over 147 days, with 95% CI. **A**: All nursing homes; **B**: nursing home I; **C**: nursing home II; **D**: nursing home III.

We observed a significant overall trend, indicating longer pain-free intervals occurring during the 147 days of observations. The pain-free interval duration was adjusted as described above (see Fig. 3). This was analog to models with three time periods. For example, in nursing home I, the corrected pain-free intervals escalated significantly (*p* < .01), from 4.7 days during the baseline to 19.2 days at T1 and 37.1 days at T2. The model equates to a substantial and significant increase of pain-free days. For nursing home II, the pain-free interval duration increase was similar. However, nursing home III did not show a significant increase between the baseline and T1 but did show a significant increase between the baseline and T2. Overall, the number of adjusted pain-free days increased significantly from the baseline (4.6 days) to T1 (15.1 days) and T2 (35.9 days). The estimated model’s Wald test was significant (*p* < .001), at χ^2^(12) = 334.18.

## Discussion

Our study of pain assessment training and bedside coaching demonstrated a substantial reduction in pain events. Pain intensity, however, remained unchanged after the intervention. In addition, the duration of pain-free intervals increased after the intervention, supporting our hypothesis. Therefore, we conclude that structured and systematic tools for the observation of those caring for people with dementia are needed. There is also a need for continuous on-site training and coaching to enable effective care for people with dementia living in nursing homes.

The on-site case study intervention applied in this research proved feasible, as it mitigated specific organizational challenges in delivering continued education and knowledge translation. A specific contextual challenge identified was the high degree of fluctuation in frontline staff—with a turnover rate of about 20% [3] — making the constant re-education of staff necessary. Furthermore, the study staff had direct links to frontline staff, allowing them to obtain feedback on implementation outcomes quickly and informally.

Regarding pain management, the results of our study seem to confirm the response certainty of the pain model [4] as a critical mechanism for frontline staff engaging in pain management. Rababa [23], based on the response certainty of the pain model, declared a need for the direct feedback and assistance of experienced clinicians to facilitate pain management in people with dementia. Tenured nurses can assist in complex cases and foster action by triggering symptom management interventions [24]. Anderson [24] suggested that rounds (i.e., regular case reviews) could complement case studies to remind frontline staff of the need for pain assessment and to foster positive reinforcement. The personal delivery of training for frontline staff has also proven promising because it allows for training content to be better tailored to the local staff roles and setting resources [11]. Anderson et al. [24] and Zúñiga et al. [25] further proposed having experienced clinicians assist local staff to enable pain and palliative care interventions. They further included rounds (i.e., regular case reviews) to remind staff of certain assessment and intervention protocols and as a means of positive reinforcement, sustaining organizational change by allowing good clinical examples to be highlighted and praised.

However, in studies such as the current one, the intervention nurse in charge of administering training and coaching faces a high degree of complexity due to having to cover the many facets of symptom management in people with dementia. For example, because people with dementia may suffer from symptoms other than pain, guidance for gerontological palliative symptom management must also be available. Furthermore, when a patient’s care plan changes, recommendations must be adapted to the local resources accordingly. Additionally, polymedication is prevalent in among people with dementia [26], and systematic, comprehensive medication reviews are rarely undertaken [3]; these require the intervention nurse to also have pharmacological knowledge.

### Limitations

The projected sample size could not be retained because the attrition rate was higher than anticipated based on previous trials in comparable populations. Furthermore, causal inference from our feasibility study is not advisable because the overall observation period was comparably short and because the design employed herein it is not as rigorous as an experimental design with clear-cut treatment and control groups. Moreover, the early presence of the coaching intervention nurse during the initial training before baseline may have shifted the intervention effect forward. As such, the baseline period may not have been entirely intervention-free, making it difficult to assess the effect of the intervention; for example, the sharp drop in pain scores in the baseline period may have been a result of this early intervention. Last, despite the promising results regarding the reduced pain frequency and longer pain-free intervals, there was no decrease in pain intensity, indicating suboptimal pain management.

## Conclusion

Personally delivered training among frontline staff, regardless of their role and educational background, may support care teams and increase team members’ confidence by making feedback more easily obtainable [6]. Direct input and assistance from an experienced clinicians helps in facilitating pain management in cases where the response certainty of the pain model for people with dementia is unclear [4, 23]. In day-to-day practice, tenured nurses are in a unique position to improve the quality of care provided through knowledge translation [27].

## Data Availability

The dataset generated is available on Zenodo from 10.5281/zenodo.6359400.

## Abbreviations

AACTT: Action Actor Context Target Time Framework
BESD: Beurteilung von Schmerz bei Demenz
CI: Confidence interval
PAINAD: Pain Assessment in Advanced Dementia Scale
SD: Standard deviation
SOAPIER: Subjective data, objective data, assessment data, plan, intervention, evaluation, recommendation
T0, T1, T2: Time periods
